# Association of socioeconomic stratification with plasmatic markers of lipoperoxidation and antioxidants in Venezuelan school-age children

**Published:** 2016-12-30

**Authors:** Nelina Ruiz-Fernández, Virgilio Bosch, Maria Isabel Giacopini

**Affiliations:** 1 Departamento de Morfofisiopatología, Escuela de Bioanálisis de la Facultad de Ciencias de la Salud, Universidad de Carabobo, Valencia, Carabobo, Venezuela.; 2 Instituto de Investigaciones en Nutrición (INVESNUT), Facultad de Ciencias de la Salud, Universidad de Carabobo, Valencia, Carabobo, Venezuela.; 3 Sección de Lipidología del Instituto de Medicina Experimental, Universidad Central de Venezuela, Caracas, Venezuela.

**Keywords:** Oxidants, antioxidants, oxidized low density lipoprotein, diet, nutritional status, child, socioeconomic factors, social class

## Abstract

**Objetive:**

To establish association between socioeconomic status and plasmatic markers of lipoperoxidation and antioxidants in Venezuelan school-age children from the middle-class and in critical poverty.

**Methods:**

Cross-sectional study with a sample of 114 school-age children (aged 7-9). The socioeconomic status, dietary intake of macro and micro-nutrients, weight, height, lipid profile, indicators of lipid peroxidation and enzymatic and non-enzymatic antioxidants were determined.

**Results:**

The daily average intake of energy, carbohydrates and vitamin A, and the percentage of energy obtained from carbohydrates was significantly higher in middle-class children compared to critical poverty children (*p* <0.05). The circulating oxidized low density lipoprotein (*p* <0.001) and the susceptibility of low density lipoproteins and very low density lipoproteins to oxidation *in vitro* (*p* <0.05) were significantly higher in middle-class children, while the critical poverty children showed significantly lower levels of Vitamin C and E in plasma (*p* <0.05). Non-enzymatic antioxidant levels were frequently deficient in both strata. The concentrations of circulating oxidized low density lipoprotein (OR: 1.09, CI _95%_: 1.016-1.179; *p*= 0.017) and Vitamin C (OR: 3.21, CI _95%_: 1.104-9.938; *p*= 0.032) were associated to the socioeconomic status independently of gender, family history of premature coronary artery disease, triglicerides, Vitamin C and E dietary intake and count of white blood cells.

**Conclusion:**

The socioeconomic status was associated to circulating oxidized low density lipoprotein and Vitamin C in Venezuelan school-age children, The results suggested the need to improve the dietary intake of antioxidants in both studied socioeconomic groups.

## Resumen

**Objetivo:**

Establecer la asociación entre la estratificación socioeconómica y marcadores plasmáticos de lipoperoxidación y antioxidantes en escolares venezolanos de clase media y en pobreza crítica.

**Métodos:**

Estudio transversal de 114 escolares con edades entre 7 y 9 años. Se determinó el estrato socioeconómico, la ingesta dietaria de macro y micronutrientes, peso, talla, perfil lipídico e indicadores plasmáticos de peroxidacion lipídica y antioxidantes enzimáticos y no-enzimáticos.

**Resultados:**

La ingesta dietaria promedio de energía, carbohidratos y vitamina A así como el porcentaje de energía obtenida de los carbohidratos fueron significativamente más elevados en los niños de clase media comparados con los niños en pobreza crítica (*p* <0.05). La concentración de lipoproteína de baja densidad oxidada circulante y la susceptibilidad de las lipoproteínas de baja densidad y muy baja densidad a la oxidación *in vitro* (*p* <0.05) fueron significativamente más elevadas en los niños de clase media, mientras los niños en pobreza crítica demostraron niveles plasmáticos de vitamin C y E significativamente más bajos (*p* <0.05). Las frecuencia de déficit de antioxidantes no enzimáticos fue elevada en ambos grupos estudiados. Las concentraciones de lipoproteína de baja densidad oxidada circulante (OR: 1.09, IC _95%_: 1.016-1.179; *p*= 0.017) y vitamina C (OR: 3.21, IC _95%_: 1.104-9.938; *p*= 0.032) se asociaron significativamente al estrato socioeconómico independientemente del sexo, historia familiar de enfermedad coronaria prematura, triglicéridos, ingesta dietaria de vitaminas C y E y contaje total de glóbulos blancos.

**Conclusión:**

La estratificación socioeconómica se asoció a las concentraciones de lipoproteína de baja densidad oxidada circulante y vitamina C en escolares venezolanos, los resultados sugirieron la necesidad de mejorar la ingesta dietaria de antioxidantes en ambos estratos socioeconómicos estudiados.

## Introduction

Cardiovascular diseases are among the primary causes of death globally. In Venezuela, heart diseases were the most frequent causes of death in 2011 ^1^. Atherosclerosis originate during infancy ^2^, the oxidation of low density lipoproteins is implied in its appearance, given that is a key factor for the generation of atheromatous plaques ^3^.

Diet is the main external factor that contributes to maintaining the body's defenses against oxidative damage ^4^ given that certain foods contain numerous antioxidants (vitamin C and E, selenium, β-carotene, etc.), although at the same time it is the source of oxidizable substrates like polyunsaturated fatty acids and prooxidant metals (iron, copper, etc.). Authors have shown differences in dietary intake in Venezuelan children of different socioeconomic status (SES) ^5^, and thus it is possible to propose that the SES could have an impact over the lipoperoxidation and antioxidants in school-age children, since they are inclined to adopt inadequate dietary patterns due to the degree of independence that they achieve during that period. 

The World Bank Report entitled "*Economic mobility and the growth of the middle class in Latin America*" published in 2013 ^6^, highlighted that after decades of stagnation, the population of middle-class in Latin America and the Caribbean has increased by 50% -103 million people in 2003 to 152 million (or 30% of the continent's population) in 2009. Applying the methodology of Graffar modified by Méndez-Castellano ^7^, the distribution of the Venezuelan population by social strata between 1982 and 2001 showed that the middle-class was on average 13.1% while the upper classes only 7.5 %; meanwhile, the percentage in critical poverty was 40.4% ^8^. Based on another method, España ^9^ reported that middle-class accounted for 35.8% for 2007. Such data have led to a genuine interest in the study of the middle-class and not only the most disadvantaged population.

To our knowledge, there has been no description of the variations of oxidative damage markers according to SES in school-age children. The data for adults is very limited, pointing to an inverse relationship between SES and lipid peroxidation in North American adults ^10^. Regarding the status of antioxidant vitamins, data indicates that when the SES is elevated, the plasma concentration of antioxidant vitamins, like ascorbic acid, increases ^11^. The most authors have compared high and low social classes, without looking at the middle class. The objective of this study was to establish association between socioeconomic status and plasmatic markers of lipoperoxidation and antioxidants in a group of Venezuelan school-age children from these two socioeconomic strata (middle-class and critical poverty).

## Materials and Methods

### Experimental design and subjects

Non-experimental transversal descriptive study that used non-probabilistic intentional sampling. The sample was conformed by 114 school-age children, both sexes, that attended six different public schools located in the Naguanagua municipality of Carabobo state, Venezuela, between 2005 and 2007. Schools were selected for their location in critical poverty or middle-class communities. Together with four additional municipalities, the Naguanagua forms the metropolitan area of the Valencia city, capital of Carabobo state, which is located in the north-central region of Venezuela. The municipality belongs to the western portion of the Coastal Mountain Range, its topography is gently sloping and flat, the temperature ranges between 22° C and 25° C ^12^. According to the 2001`s Census, it had a population of 132,368 habitants, with a population density of 704.1 inhabitants/km^2^ and 23% of households was classified as poor ^12^ ^,^ ^13^. The town bases its economy on the intensive commercial activity ^12^, receiving foods produced within and outside the Carabobo state. The main agricultural products produced in that state are coffee, sugar cane, bean, corn and orange. In Carabobo State predominant poultry industry, cattle and pig; it also receives marine fisheries products and has a well developed agroindustrial sector ^14^. 

The inclusion criteria in the sample were: age between 7 and 9 years old, to belong the middle-class (MC) or critical poverty (CP), apparently healthy, without medical diagnosis of any acute infectious-inflammatory process, no personal history of neurological disease, diabetes mellitus, arterial hypertension or kidney disease. All Helsinki Declaration accords were fulfilled, obtaining a signed informed consent from the children's parents or guardians. 

The research protocol was informed and approved by the superintendent of each school. A registry of all enrolled students aged 7 to 9 years old was obtained at the time of selection. Notification was sent to the parents describing the study protocol and the blood collecting conditions. Through a survey to parents applied by researchers, data of children (socioeconomic status, personal medical history and current illnesses, family medical history about premature coronary artery disease and drug treatment) were obtained. Children who on the day of the evaluation showed symptoms of infectious diseases were excluded, as well as those that were being treated with steroids or those that did not attend school due to illness in the seven days prior to the day of the evaluation. For each child that did not meet the inclusion criteria or that did not attend, another one was brought in until finishing the evaluation of at least 50 children for each SES studied.

### Socioeconomic, anthropometric and dietary evaluation

The SES was determined through the Graffar`s Method, modified for Venezuela by Hernán Méndez Castellano^7^, which evaluates the profession of the head of the family, education level of the mother, the main source of income of the family and condition of the home. The sum of the four evaluated variables defined the stratum of child, according to the following scale: 10-12 points for MC and 17-20 points for CP. In this method, the SES is inversely proportional to the obtained punctuation.

Anthropometric nutritional status and dietary intake were determined due that they were considered intervening/confusing variables. Following the established protocols ^15^, weight and height of the child were measured using a digital balance (Tanita brand, precision 0.1 kg) and metric tape adhered to a wall (precision 0.1 cm). Body mass index (BMI) was calculated by dividing the body weight (in kilograms) by the height (in meters) squared (kg/m^2^). The z score values for height-, weight- and BMI-for-age relative to the WHO 2007 reference were calculated using WHO AnthroPlus (version 1.0.4) ^16^.

The daily intake of energy, carbohydrates, fats, proteins, iron, vitamins C, A and E, were estimated through a 24-hour dietary recall; when the child was ≥8 years old, the questionnaire was aimed firstly at the child, confirming the information with the mother to obtain higher precision in the acquired data. To estimate the size of the food rations, common measuring tools and modeled foods were presented. Daily intake of nutrients was estimated using the Venezuelan Food Composition Table ^17^, along with other nutritional information databases ^18^ ^,^ ^19^ for those foodstuffs and nutrients not included in the Venezuelan Table. Whenever these were not found in such tables, they were substituted with the most similar foodstuffs and/or preparations. The percentages of energy provided by carbohydrates, proteins and fats were calculated.

### Blood extraction and laboratory analysis

After a 12-14 hour fast, 12 mL of blood was extracted through venipuncture at the elbow joint, and distributed in tubes containing EDTA and heparin; the tubes were kept away from sunlight. A aliquot of total blood was taken to measure the glutathione peroxidase (GPx) activity and to perform an automated complete heme profile (hemoglobin concentration, count of total white blood cells, eosinophils and lymphocytes). The remaining blood was centrifuged (10 min at 1,000 g) to extract the erythrocyte portion to determine the superoxide dismutase (SOD) activity, and plasma to measure thiobarbituric acid reactive substances (TBARS), *in vivo* circulating oxidized low density lipoproteins (OxLDL), susceptibility of the plasma VLDL and LDL to oxidation *in vitro,* total antioxidant capacity (TAC), vitamins A, E and C, total cholesterol (TC), triglycerides, (TG), high density lipoprotein bonded cholesterol (HDLc) and C-reactive protein (CRP).

### Lipoperoxidation markers

TBARS measurement was performed using a colorimetric method ^20^ ^,^ ^21^. OxLDL was measured with a two-point solid phase enzyme immunoassay with the murine specific monoclonal antibody 4E6 (Mercodia AB, Sweden), using a Tecan Sunrise ELISA Reader. To determine the susceptibility of the plasma VLDL and LDL to oxidation *in vitro*, the lipoproteins were separated from the plasma using the density adjustment technique and serial centrifugation, and TBARS were measured three hours after incubating the lipoproteins with copper following the procedure previously described ^22^.

The normative range for OxLDL given by manufacturers in the kit is 26-117 U/L; in healthy adults, range for TBARS is 0.27-1.28 (mol/L for women and 0.35-1.10 (mol/L for men ^23^. Since not available normal values in Venezuelan children, lipoperoxidation markers were considered elevated when they were above the 90th percentile (calculated in the total group of evaluated children). 

### Antioxidant markers

Plasma TAC, activity of SOD and GPx were measured using commercial kits (Randox Laboratories Ltd., United Kingdom). Vitamin C was quantified applying a colorimetric method ^24^. Spectrophotometric readings of these antioxidant indicators and TBARS were obtained with a Stat Fax Millenium III. 

The normative ranges given by manufacturers in the kit are: 1.30-1.77 mmol/L for TAC, 1102-1601 U/g Hb or 164-240 U/mL for SOD, 27.5-73.6 U/g Hb or 4171-10881 U/L for GPx. TAC, SOD and GPx were considered low when they were below their 10th percentile calculated in the total group, because not available normal values ​​were found in Venezuelan children.

Vitamins A and E were analyzed through high performance liquid chromatography, using the methodology of Bieri *et al*. ^25^, with a Hewlett Packard 1050 chromatograph, under the following conditions: reverse phase column of octadecyl silica as the stationary phase (Zorbax Eclipse, XDB-C8 x 150 mm, 5 mm) and a 95:5 methanol:water mixture as mobile phase. The flow rate was maintained at 1.5 mL/min. The vitamin E/TC index was calculated. As antioxidants, vitamin C, A and E values and the vitamin E/TC index were deficient when they were lower than 0.9 mg/dL, 74.4 (g/dL, 1.3 mg/dL and 4.85 umol/mmol ^26^.

### Other tests

The level of hemoglobin allowed quantify the activity of antioxidant enzymes while CRP and absolute counts of white blood cells, eosinophils, and lymphocytes were used as indicators of infectious-inflammatory process, since these can induce lipoperoxidation. CRP was detected in plasma using a commercial qualitative test based on latex particle agglutination (Teco Diagnostics, USA). Only two children showed positive CRP, however the measured indicators in these children were not significantly different than those obtained in children with negative CRP, and thus they were not excluded from the study. 

The concentrations of TC, TG, and HDLc were assayed using commercial kits (Laboratorio CienVar, Venezuela), through enzymatic colorimetric methods with Stat Fax Millenium III. These lipid markers were evaluated because its absolute levels can influence the degree of lipid oxidation.

### Statistical analysis

Central and dispersion measures and absolute and relative frequencies were calculated. The Kolmogorov-Smirnov test was used to determine whether the variables followed a normal distribution. An unpaired student t-test or Mann-Whitney Test U test was used, depending on the case, to compare the biochemical variables according to age, gender and SES. Chi-squared test to associate the SES with alterations of biochemical indicators. Correlation analysis (Pearson coefficient or Spearman's Rho, depending on the case) to determine whether the biochemical indicators correlated with the SES and dietary intake.

The associations between SES and biochemical indicators of lipoperoxidation and antioxidants were reevaluated by binary logistic regression, adjusting for following covariates: age, gender, family history of premature coronary artery disease, BMI z-score, total count of white blood cells, TC, TG and vitamin dietary intake. A stepwise selection method was chosen for the introduction/removal of the variables in the regression model. All analysis was executed using the PASW Statistics Multilanguage version 18.0 package, considering a significance level of <0.05.

## Results

Houndred fourteen schoolchildren were evaluated (55 girls and 59 boys) with an average age of 8.0 ±0.8 years old, where 37.7% were seven years old, 28.9% were eight years old and 33.3% were nine years old. According to the modified Graffar´s method, 53.5% (30 girls and 30 boys) belonged to MC, and 46.5% (25 girls and 29 boys) was in CP. The distribution of the SES was not associated with age or gender. Middle-class children exhibited significantly higher values of average Graffar´s score, age, weight z-score, BMI, BMI z-score, TC and TG values, as well as lower concentrations of HDLc, compared to children in CP (Table 1). No significant difference was observed in the frequency of children with family history of premature coronary artery disease according to the SES.

**Table 1:** Socioeconomic, clinical and general laboratory variables in the total group and categorized by socioeconomic status*

Variable	Total Group	Socioeconomic Status	
		Middle-Class	Critical Poverty
Graffar´s Score (points)	14.0±3.0	11.3±1.2^§^	17.1±0.3
Age (years)	8.0±0.8	7.8±0.8^¶^	8.2±0.8
Weight (Kg)	27.7±6.4	28.7±7.1	26.6±7.0
Weight z-score	0.11±1.12	0.34±1.06^†^	-0.16±1.14
Height (cm)	129±7.6	130±8.2	129±7.0
Height z-score	0.03±0.93	0.13±0.86	-0.09±1.01
Body mass index (kg/m^2)^	16.4±2.4	16.9±2.7^†^	15.8±1.9
Body mass index z-score	0.08±1.22	0,32±1.26^†^	-0.19±1.14
Total cholesterol (mg/dL)	142.6±30.2	148.3±34.6^‡^	133.5±23.8
Triglycerides (mg/dL)	73.8±34.4	79.1±35.7^†^	66.6±31.6
High density lipoprotein-cholesterol (mg/dL)	34.9±9.0	33.3±8.4^¶^	36.7±9.2
Hemoglobin (g/dL)	13.0±0.8	13.1±0.8	13.0±0.9
White blood cells (cells/mm^3)^	6,180±1,704	6,121±1,880	6,247±1,489
Eosinophils (cells/mm^3)^	462±451	404±320	530±562
Lymphocytes (cells/mm^3)^	2,732±958	2,715±922	2,750±1,005
Children with a family history of premature coronary artery disease n (%)	3 (2.6)	2 (1.8)	1 (0.9)

*n= 114 (61 from middle-class, 53 from critical poverty). Arithmetic mean±standard deviation.

†*p* <0.05, t-student between socioeconomic strata.

‡ p <0.01, t-student between socioeconomic strata.

¶ p <0.05, Mann-Whitney test between socioeconomic strata.

§ p <0.01, Mann-Whitney test between socioeconomic strata.

The daily average intake of energy, carbohydrates and vitamin A, as well as the percentage of energy provided by carbohydrates were significantly higher in MC children compared to children in CP (Table 2). 

**Table 2:** Dietary intake of energy and nutrients in the total group and according to socioeconomic status*

Nutrients	Total Group		Middle-Class		Critical Poverty	
	Mean±SD	Median	Mean±SD	Median	Mean±SD	Median
Energy (kcal/day)	1,547.5±393.0	1,570.9	1,620.0±312.4^†^	1,628.0	1,464.0±458.0	1,379.6
Proteins (g/day)	57.8±19.2	57.0	60.3±19.6	60.1	55.1±18.5	56.2
%Energy as proteins	14.8±4.9	14.4	14.9±4.7	14.8	15.1±5.1	16.3
Fat (g/day)	46.1±19.7	42.5	48.6±18.2	45.5	43.1±21.1	41.5
%Energy as fat	26.7±11.4	24.3	26.8±10.2	25,2	26.4±12.7	27.1
Carbohydrate (g/day)	245.0±61.1	252.7	270.6±46.8^†^	277.9	217.7±72.5	218.0
%Energy as carbohydrate	63.3±15.7	65.3	66.8±11.8^†^	68.6	59.5±19.5	59.6
Iron (mg/day)	13.6±5.0	13.0	14.1±5.0	13.4	13.0±5.0	12.5
Vitamin A (retinol equivalent/day)	1,041.1±806.4	777.7	1,193.3±89.0^‡^	1,055.3	866.0±662.3	697.6
Vitamin C (mg/day)	58.5±65.1	36.7	65.6±68.2	39.9	50.3±61.0	27.8
Vitamin E (mg/day)	5.1±3.4	4.3	5.4±3.4	4.5	4.8±3.4	4.0

* n=114 (61 from middle-class, 53 from critical poverty).

† *p* <0.05, t-student between socioeconomic strata.

‡ *p* <0.05, Mann-Whitney test between socioeconomic strata.

None of the studied biochemical indicators differed with age. Only plasma vitamin E was significantly higher in girls than in boys (0.56 ±0.14 vs. 0.51 ±0.12 mg/dL; *p* <0.05). Lipoperoxidation markers did not correlate significantly with dietary intake. Only vitamin C in plasma was correlated with its dietary intake (r= 0.221; *p* <0.05).

Plasma concentrations of OxLDL and vitamins C and E, as well as the susceptibility of the plasma VLDL and LDL to oxidation *in vitro* were significantly higher in MC children (Table 3). In contrast, children in CP showed significantly lower levels of vitamins C and E in plasma.

**Table 3:** Indicators of prooxidant-antioxidant balance in total group and categorized by socioeconomic status*

Indicator	Total Group	Socioeconomic Status	
		Middle-Class	Critical Poverty
Prooxidant			
Thiobarbituric Acid Reactive Substances (nmol/mL)	0.88±0.5 (114)	0.92±0.6 (63)	0.84±0.2 (51)
Circulating oxidized low density lipoprotein (U/L)	43.9±13.8 (82)	50.2±13.3^‡^ (41)	37.6±11.2 (41)
Susceptibility to oxidation of very low density lipoproteins (nmol/mg protein)	8.3±6.0 (77)	9.6±5.8^†^ (41)	6.9±5.9 (36)
Susceptibility to oxidation of low density lipoproteins (nmol/mg protein)	1.3±1.2 (79)	1.6±1.3^¶^ (41)	1.0±0.9 (38)
Antioxidant			
Superoxide Dismutase activity (U/mL)	192±42 (114)	195±34 (63)	189±50 (51)
Superoxide Dismutase activity (U/g Hemoglobin)	1,479±333 (114)	1,497±281 (63)	1,459±386 (51)
Glutathione Peroxidase activity (U/L)	7,958±1,883 (114)	8,185±1891 (63)	7,698±1858 (51)
Glutathione Peroxidase activity (U/g Hemoglobin)	61.0±14.6 (114)	62.2±14.5 (63)	59.6±14.9 (51)
Vitamin C (mg/dL)	0.98±0.43 (114)	1.06±0.42 (63)	0.88±0.42^_† (51)_^
Vitamin A (μg/dL)	61.6±23.8 (114)	64.0±24.2 (63)	58.8±23.2 (51)
Vitamin E (mg/dL)	0.43±0.14 (114)	0.46±0.14 (63)	0.40±0.12^_† (51)_^
Vitamin E/total cholesterol Index (μmol/mmol)	2.77±0.83 (114)	2.78±0.79 (63)	2.77±0.90 (51)
Total Antioxidant Capacity (mmol/L)	1.34±0.15 (114)	1.35±0.14 (63)	1.34±0.17 (51)

* n in parentheses. Arithmetic mean±standard deviation.

† p <0.05, t-student between socioeconomic strata.

‡ p <0.001, t-student between socioeconomic strata.

¶ p <0.05, Mann-Whitney test between socioeconomic strata.

The frequencies of children with elevated TBARS, OxLDL and susceptibility of the plasma VLDL and LDL to oxidation *in vitro* were about 9.6%, 9.8%, 19.5% and 6.3%, respectively. Only the percentage of with circulating OxLDL above the 90th percentile was significantly associated with that SES, placing all these cases in the MC (Chi^2^= 8.865; *p* <0.01). The Table 4 shows the frequency of children with deficits in antioxidant defense markers in the total sample and according to SES, we observed that vitamin C, A and E deficits were frequent. The percentage of children with lower vitamin C antioxidant levels (0.9 mg/dL) was significantly higher among children in CP (Chi^2^= 8.815; *p* <0.01). Plasma vitamin E concentration in all evaluated children did not reach a value to provide efficient antioxidant protection (1.3 mg/dL) and the number of children showing vitamin E deficit continued to be considerable when the vitamin E/TC index was analyzed (98.2%), where there were no differences between socioeconomic strata studied. 

**Table 4:** Frequency of children with deficiency of blood antioxidant defense in the total group and according to socioeconomic status

Antioxidant Alteration	Total Group	Socioeconomic Status	
		Middle-Class	Critical Poverty
Low erythrocyte superoxide dismutase activity	9.6	4.9	15.1
Low blood glutathione peroxidase activity	11.4	13.1	9.4
Low serum Vitamin C	47.4	34.4	62.3^*^
Low serum Vitamin A	50.9	47.5	54.7
Low serum Vitamin E	100	100	100
Low serum Vitamin E/total cholesterol Index	98.2	98.4	98.1
Low serum total antioxidant capacity	9.6	8.2	11.3

n=114 (61 from middle-class, 53 from critical poverty).

Data expressed as percentages calculated based on the total number of children in every socioeconomic stratum.

* *p*= 0.003, Chi-squared Test between socioeconomic strata.

The SES was positively associated with circulating OxLDL (r= 0.459; *p* <0.001), oxidative susceptibility of VLDL (r= 0.229; *p* <0.05) and LDL (r= 0.282; *p* <0.05) as well as the plasma levels of Vitamin C (r= 0.222; *p* <0.05) and E (r= 0.200; *p* <0.05). 

The Table 5 shows results of binary logistic regression. The SES was associate to circulating OxLDL independently of age, gender, family history of premature coronary artery disease, BMI z-score, TG and white blood cells count, but not of total cholesterol. Only plasma vitamin C was associated to the SES independently of gender, family history of premature coronary artery disease, TG, vitamin C and E dietary intake, count of white blood cells, but not of age, BMI z-score and total cholesterol.

**Table 5:** Model of the association between indicators of prooxidant-antioxidant balance and socioeconomic status

Indicator	ß(SE)	OR (CI95%)	p
Prooxidant			
Circulating oxidized LDL	0.090 (0.038)	1.09 (1.016-1.179)	0.017
Total cholesterol	0.034 (0.013)	1.03 (1.009-1.061)	0.009
Antioxidant			
Vitamin C	1.166 (0.545)	3.21 (1.104-9.938)	0.032
Age	-0.572 (0.262)	0.565 (0.338-0.943)	0.029
Body mass index z-score	0.521 (0.187)	1.68 (1.167-2.431)	0.005
Total cholesterol	0.020 (0.009)	1.02 (1.003-1.037)	0.022

Binary logistic regression (crtical poverty as reference stratum). Only variables remaining in the model are shown. The association was adjusted for age, gender, family history of premature coronary artery disease, body mass index z-score, absolute counts of white blood cells, total cholesterol and triglycerides, and vitamin dietary intake (only for antioxidant indicators).

SE: standard error of the b coefficient; CI: confidence interval (lower limit, upper limit).

## Discussion

According to the "current" socioeconomic status model, socioeconomic experiences at some point of the life cycle may have an influence on health, which suggests that current life conditions have an immediate impact on health ^27^. Own dynamic nature of the socioeconomic variables and specific geographical and environmental contexts impose a need to periodically evaluate the influence that the SES has on health in different population groups. Based on these considerations, this study examined the impact socioeconomic stratification on plasmatic markers of lipoperoxidation and antioxidants in Venezuelan schoolchildren.

Anthropometric nutricional status and dietary intake are directly involved in the pro-oxidant antioxidant balance, so in this study were evaluated. As expected weight z-score, BMI and BMI z-score were signicantly higher in MC children, although the mean z-scores were within normal in both strata studied. Schoolchildren in CP showed significantly lower average dietary intake of energy, carbohydrates and vitamin A than those registered in MC children. Such results coincide with observations made by other authors in Caracas and Valencia, Venezuela ^5^ ^,^ ^28^ and European countries ^29^. Likewise it is consistent with a systematic review of social differences related to dieteria intake, specifically in low- and middle-income countries ^30^.

In this study different lipoperoxidation markers were measured. The large variation in TBARS assays due to diverse factors precludes assigning diagnostic reference standard criteria, however , the values ​​found in the total group and the CP group lied within the 10^th^-90^th^ percentiles of TBARS determined in healthy adults ^23^, while the value of the MC group was slightly higher. Souki *et al*. ^31^, in children (aged 6-9) of Maracaibo city, found mean values of malondialdehyde (main substance reactive to thiobarbituric acid in plasma) that were practically identical to those observed in this study. 

This the first report of OxLDL levels in Venezuelan children according to the reviewed literature. Using the normative range given by the manufacturers for used kit (Source: Mercodia Oxidized LDL Elisa), the concentrations of OxLDL in total sample and studied groups were within expected but lower than that described by Kelly *et al*. ^32^, in North American children of normal BMI. According to the 90th percentile calculated in the total group, 10% of children had elevated levels of OxLDL. This frequency is about half of that found in a group of Venezuelan women with overweight from the same region of the country ^33^. Likewise, the only previous report about susceptibility to oxidation *in vitro* of VLDL and LDL in Venezuelan children was conducted by our research group ^22^. The degree of oxidation of VLDL and LDL observed in this study was lower than that shown in the first study, a difference that could be attributed to the variability of data in the first study.

Interestingly and contrary to expectations, the MC children showed higher lipoperoxidation, with higher OxLDL levels and higher susceptibility of VLDL and LDL to oxidation *in vitro*. Likewise, all of the students that showed values of OxLDL over the 90th percentile belonged to MC, and the positive association of this indicator with the SES was independent age, gender, family history of premature coronary artery disease, BMI z-score, TG and total counts of eosinophils and lymphocytes. There is little information about the influence the SES would have on lipoperoxidation. Janicki-Deverts *et al*. ^10^, published data that did not coincide with our results, reporting an inverse relationship between the socioeconomic level and the levels of F2-isoprostanes and γ-glutamyl transferase in young North American adults (18-30 years old). To our knowledge this is the first study evaluated the association between these markers and the SES in Venezuelan children. Regardless of that our observations must be confirmed by larger studies, this investigation provides evidence for the first time that the oxidative damage on lipids is related to socioeconomic level in Venezuelan schoolchildren. This finding could have potential implications in the mortality profile that MC children could develop in the future as adults, since it has been informed a positive association between the levels of circulating OxLDL (determined by the 4E6 antibody) and the intima-media thickness of the carotid arteries ^34^, a marker of subclinical atherosclerosis. 

It is not possible to explain the differences that were observed in lipoperoxidation based on the amount of fat ingested in the studied strata. However, it is important to remember that it is not just the amount but also the type of fat consumed that can affect the susceptibility of lipoproteins to oxidation, given that unsaturated fatty acids (FA) are the substrate of these oxidation reactions. High SES has been significantly associated with higher fat intake (total fat, cholesterol, polyunsaturated FAs, saturated FAs and monounsaturated FAs&) in low- and middle-income countries, for example relative differences for high compared with low SES range 6% for polyunsaturated FA ^30^. There is no data in Venezuela about the pattern of dietary fatty acid that is consumed in different socioeconomic strata. In Colombian population the use of sunflower oil, the main fat source used in cooking, has been positively associated to the SES ^35^; a similar situation was proven in Costa Rica with soybean oil ^36^. It is recognized that the most consumed oils by the Venezuelan population are from corn, sunflower and soybean ^37^. Such oils have significant levels of linoleic acid (C18:2 n-6), which is associated with an increase of the oxidation susceptibility of the LDL ^38^. In this study the concentration of TBARS was measured three hours after incubation of VLDL and LDL with copper, so that the final stage of the reaction was evaluated, which is in direct relation with the unsaturated FA content of the lipoprotein, this allows to propose that the greater degree of in vitro oxidation of LDL and VLDL and higher levels of circulating OxLDL observed among MC children probably are explained through the enrichment of their LDL and VLDL with polyunsaturated FA. Further studies should evaluate this hypothesis.

Similary to TBARS, the large variation in assays and their expression across the literature precludes assigning reference values for measured enzymatic antioxidants. Our children showed SOD and GPx activities similar to those reported in Guatemalan low-income preschoolers ^39^. No differences were found in enzymatic antioxidants according SES, but the levels of non-enzymatic antioxidants like vitamins C and E were higher in MC children. Such findings coincide with observations made by other authors in adult individuals ^11^ ^,^ ^40^. Almost half of the studied children did not have a acceptable level of plasma vitamin C as an antioxidant, being this finding significantly more frequent in children in CP. The vitamin C not only sweeps away effectively reactive species attacking LDL particle, but also regenerates the oxidized form of vitamin E ^41^. The evidence suggests that intake of vitamin C of studied children was not sufficient to obtain the optimal levels of antioxidants suggested by Gey ^26^. This is concerning since the abundance of vitamin C in fruits and vegetables found in Venezuela. In low- and middle-income countries, high SES is associated with higher fruit and/or vegetable consumption, diet quality and diversity ^30^, which it is essential for to get the sources of the different antioxidant vitamins.

Serum concentrations of vitamin E in the total sample and the studied groups were between 9 y 12 μmol/L, values that are considered marginal from the point view "nutrition" ^42^, coinciding with the findings by Soto-Méndez in poor Guatemalan children ^39^. Numerous reports worldwide have shown that such concentrations are frequently reported in children ^42^, aggravated when considering the antioxidant function of vitamin E. Thus, all schoolchildren evaluated were vitamin E deficient from an "antioxidant" point of view, and according to the vitamin E/TC index 98.2% of children was in deficit. Carias *et al*. ^43^, reported similar findings in young pre-college subjects in Caracas. These observations deserve special attention because of its implications in the development of atherosclerotic lesions from childhood, since apha-tocopherol stops the chain propagation of lipoperoxidation in LDL ^37^. Traber and Sies ^44^ ^)^ estimated that to maintain a plasma tocopherol level of 1.3 mg/dL it is required an intake of 15 to 30 mg/day of vitamin E, which could be accomplished through foods rich in this vitamin. Such dietary intake is at least three times higher than what was consumed by children in this study, highlighting once again the need to promote the regular intake of foods that allow raising vitamin E levels in the plasma of Venezuelan children. Finally, plasmatic levels of vitamins C, A and E, suggested by Gey ^26^ as adequate from an antioxidant point of view, are standardized based on the concentrations of lipids in plasma normally observed in adults (TC= 220 mg/dL and TG= 100 mg/dL), which are clearly higher than those normally found in Venezuelan schoolchildren, so it is difficult to interpret the results.

When the results of this investigation are analized under an integrated focus, highlights that MC children not only showed higher lipoperoxidation but also more antioxidant defense compared to CP children. This situation would reflect the complex relationship between socioeconomic level and the oxidant/antioxidant status in school-age children, as well as the variety of factors that could mediate in it, where an unbalanced dietary intake in quality and quantity would probably be one of the more relevant factor. In the group of studied schoolchildren, the SES was associated to plasma levels of OxLDL and vitamin C independently of gender, family history of premature coronary artery disease, triglicerides, Vitamin C and E dietary intake and count of white blood cells, this finding has two main implications. One relates directly to demonstrating the impact of the socioeconomic stratification over prooxidant-antioxidant balance in school-age children. The second would be of practical nature, indicating that the studies that involving these markers must consider, at least in the evaluated age group, the SES as a relevant variable in sample selection and data processing.

Finally, it is necessary to point out that this study has limitations. Firstly, the results of the evaluation of dietary intake must be considered with caution given that the intake at each day of the week was not equitably represented because that only a 24-hour recall was collected. Secondly, the impossibility of establishing a causal relationship between the SES and the evaluated markers, due the transversal design of the study. The third limitation arises from the inclusion criteria in this regard, it is likely that the characteristics of the population that does not attend educational institutions are different, especially in the case of children living in critical poverty. The latter may explain why no a greater number of significant differences were found between the studied strata, as was expected. 

## Conclusion

The socioeconomic status was associated to *in vivo* circulating oxidized low density lipoproteins and Vitamin C in Venezuelan school-age children, demonstrating higher lipoperoxidation in middle-class subjects and lower vitamin C and E concentrations in plasma in critical poverty subjects. An important percentage of children from both socioeconomic strata showed plasma vitamin values below the acceptable level from an antioxidant point of view, which would suggest the need to improve the consumption of foods rich in antioxidants in both groups.
